# Glenohumeral Stability During External Rotation in Patients Treated With the Latarjet Procedure: A Two‐Year Dynamic Radiostereometric Study

**DOI:** 10.1002/jor.70258

**Published:** 2026-08-09

**Authors:** Josephine Olsen Kipp, Emil Toft Petersen, Maiken Stilling, Anna Zejden, Rikke Jellesen Åberg, Thomas Falstie‐Jensen, Theis Muncholm Thillemann

**Affiliations:** ^1^ AutoRSA Research Group, Orthopaedic Research Unit Aarhus University Hospital Aarhus N Denmark; ^2^ Department of Clinical Medicine Aarhus University Aarhus Denmark; ^3^ Department of Orthopaedic Surgery Aarhus University Hospital Aarhus N Denmark; ^4^ Department of Radiology Aarhus University Hospital Aarhus N Denmark

**Keywords:** anterior shoulder instability, biomechanics, glenohumeral joint kinematics, glenoid bone loss, the Latarjet procedure

## Abstract

The Latarjet procedure is widely used for anterior shoulder instability with glenoid bone loss, yet its influence on glenohumeral joint (GHJ) kinematics during functional movement remains unclear. This study evaluated GHJ kinematics during active external rotation compared with the contralateral shoulder preoperatively and at 1‐ and 2‐year follow‐up using dynamic radiostereometry (RSA) and CT‐derived 3D bone models registered to the radiographs. Patient‐reported outcomes were assessed using the Western Ontario Shoulder Instability Index (WOSI). Preoperatively, the injured shoulder showed a tendency toward a more anterior (up to 1.5 mm, CI −0.3–3.3) and inferior (up to 1.1 mm, CI −0.3–2.5) humeral head position. At 1 year postoperatively, the humeral head was more posterior (up to 1.8 mm, CI 0.8–2.9) and superior (4.0 mm, CI −4.1–12.1) compared with preoperatively, with an additional posterior shift at 2 years (1.5 mm, CI 0.4–2.6). Postoperative kinematics did not differ from the healthy shoulder. Contact area decreased preoperatively by up to 121.7 mm^2^ (CI 57.1–186.3) and increased by up to 110.8 mm^2^ (CI 42.3–179.3) at 2 years. WOSI improved from 55% (CI 49–61) preoperatively to 36% (CI 25–48) at 1 year and 27% (CI 17–36) at 2 years. The Latarjet procedure resulted in GHJ kinematics comparable to the healthy shoulder during external rotation. Postoperatively, kinematics shifted posteriorly and superiorly with improved WOSI scores over 2 years.

**Clinical Significance:** Near‐normal GHJ kinematics during external rotation are achieved within 2 years, supporting improved shoulder function in anterior instability following the Latarjet procedure.

## 1 Introduction

The Latarjet procedure is the preferred treatment for anterior shoulder instability, particularly in cases involving significant glenoid bone loss [1, 2]. While the efficacy of the Latarjet procedure in preventing recurrent dislocations is well‐documented, its effect on GHJ kinematics in patients remains limited and has primarily been examined in experimental settings [3, 4, 5]. Most studies have assessed GHJ kinematics in static positions, typically with the arm abducted and in end‐range external rotation, and report no significant postoperative differences compared to the healthy shoulder [3, 5, 6, 7, 8, 9, 10]. However, such assessments do not capture the complex, dynamic behavior of the GHJ during movement and represent only a small part of functional shoulder use, particularly since these positions are rarely achieved in daily life.

To overcome these problems, three studies have examined the GHJ kinematics during isometric muscle contraction in an abducted and externally rotated position or during an external rotation motion [3, 4, 11]. However, these studies are limited by passive or constrained muscle activation and analyses restricted to unidirectional translation without including the preoperative condition. These studies reported altered kinematic patterns compared with the healthy shoulder. Moreover, a more posterior and superior humeral head position following the Latarjet procedure has been described, raising concerns about potential overcorrection, which may affect joint function and long‐term outcomes [3, 4, 12]. Consequently, 3D in vivo GHJ kinematics during active external rotation before and after the Latarjet procedure remain insufficiently characterized.

Several recording methods may be used to assess GHJ kinematics. We have previously evaluated GHJ kinematics using dynamic radiostereometric analysis (RSA), due to the high precision and non‐invasive nature [11, 13, 14, 15, 16].

The aim of this study was to investigate GHJ kinematics during active external rotation in patients before, 1 year, and 2 years after shoulder surgery with the Latarjet procedure in comparison to the contralateral healthy shoulder. The hypothesis was that glenohumeral joint kinematics would be restored to those of the contralateral healthy shoulder and remain stable during follow‐up.

## 2 Methods

### 2.1 Study Design and Patients

This prospective cohort study included 19 patients diagnosed with unilateral traumatic anterior shoulder instability and scheduled for the Latarjet procedure, recruited between February 2022 and March 2023 at Aarhus University Hospital, Denmark. The inclusion criteria were: (1) age 18–40 years, (2) a healthy contralateral shoulder, and (3) ≥ 10% glenoid bone loss assessed by the best‐fit circle method on CT [17]. Exclusion criteria included bilateral or multidirectional instability or alcohol or drug abuse.

The Danish Data Protection Agency (journal no 1‐16‐02‐344‐21) and the Central Denmark Region Committees on Health Research Ethics (journal no 1‐10‐72‐298‐21) approved the study. Written informed consent was obtained from all participants. The study adhered to the Declaration of Helsinki and was reported in accordance with the STROBE guidelines [18].

### 2.2 Test Setup

An open Latarjet procedure was performed by experienced shoulder surgeons as described by Young et al. [19]. Glenohumeral kinematics were assessed preoperatively and at 1‐ and 2‐year follow‐up using dynamic RSA (Adora RSA system, NRT X‐ray, Denmark), a biplanar X‐ray technique with CT‐based 3D tracking of the bone models (humerus and scapula) during external rotation [12, 13, 20]. Patient‐specific bone volume models were extracted from the CT scans using automated graph‐cut segmentation [21, 22]. These models were then registered to the calibrated RSA images (RSAcore, Leiden, the Netherlands) using a two‐step optimization procedure (AutoRSA software, AutoRSA Research Group, Aarhus, Denmark). Initial alignment was performed in half resolution utilizing a global optimizer (controlled random search algorithm with local mutations), followed by a local optimizer (Nelder–Mead Simplex). A final high‐resolution optimization was subsequently performed, excluding the models' surroundings [23].

**FIGURE 1:**
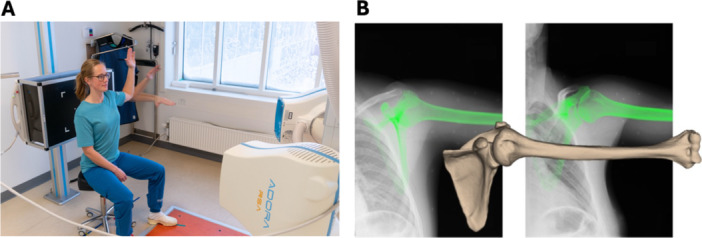
(A) Two synchronized X‐ray tubes recorded continuously while patients performed an approximately external rotation from horizontal to maximal external rotation in an upright posture with the elbow in 90° flexion and the shoulder abducted to 90°. (B) Illustration of the 3D bone models and their projections (green) aligned with the RSA recording.

Both shoulders were assessed preoperatively. At 1‐ and 2‐year follow‐up, only the operated shoulder was assessed. Patients performed active external rotation from horizontal to maximal external rotation and back again in an upright posture with the elbow in 90° flexion and the shoulder abducted to 90°. Motion was performed over approximately 1–2 s and captured at 10 Hz (Figure 1).

### 2.3 Glenohumeral Joint Kinematics

**FIGURE 2:**
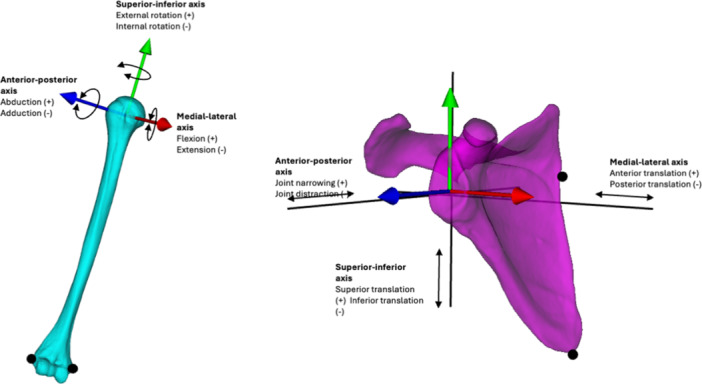
The anatomical landmarks are marked with a black dot on the origin of the coordinate system. The humerus (cyan): the superior‐inferior axis was defined by the midpoint between the medial and lateral epicondyles, and the center of a best‐fit sphere defined the glenohumeral center to the articulating part of the humeral head. The anterior‐posterior axis was the line perpendicular to the plane formed by the epicondyles and the glenohumeral center. The medial‐lateral axis was the cross‐product of the anterior‐posterior and superior‐inferior axes. The scapula (purple): the medial‐lateral axis was defined as the line between the trigonum spinae and the glenoid center, defined by a best‐fit circle to the inferior part of the glenoid. The superior‐inferior axis was defined by an orthogonal projection from the medial‐lateral axis toward the inferior angle of the scapula. The cross‐product of the medial‐lateral axis and the superior‐inferior axis was defined as the anterior‐posterior axis.

Anatomical landmarks were manually assigned to the preoperative bone models, and GHJ kinematics were described using Cardan angles (abduction‐flexion‐external rotation) to assess motion in six degrees of freedom, with the humerus analyzed relative to the scapula (Figure 2) [24, 25]. Three rotations: abduction(+)/adduction(−), flexion(+)/extension(−), and external rotation(+)/internal rotation(−) and two translations: anterior(+)/posterior(−), superior(+)/inferior(−) were evaluated. The contact area was defined as the area between the humerus and glenoid where the distance was less than 6 mm, based on the assumption of a maximum 2–3 mm cartilage thickness on the humerus and glenoid [26, 27]. The standardized test setup enabled the rotation to be expressed as a normalized cycle ranging from 1 to 100, where 1 corresponded to the starting position, 50 to the end of external rotation, and 100 to the end of internal rotation.

### 2.4 Patient‐Reported Outcome Measures

Preoperatively, and at 1 and 2 years postoperatively, patients completed the Western Ontario Shoulder Instability Index (WOSI) questionnaire (range: 0–2100, with 0 representing the best outcome). The minimal clinically important difference (MCID) is 220 points for shoulder instability [28]. The WOSI score was transformed into a percentage score (0%–100%, with 0 being the best), resulting in an MCID of 10.4%.

### 2.5 Statistics

Statistical parametric mapping (SPM) (spm1d.org, v.0.4.18, Python Software Foundation v.3.10) using a two‐sided paired Student's *t*‐test was employed to evaluate and compare the GHJ kinematics during the trajectory of the GHJ external rotation with a significance level of 5%. Assumptions with random, homologous, and normally distributed data were tested.

### 2.6 Sample Size

The sample size calculation was based on humeral head translation in patients operated with the Latarjet procedure and in healthy subjects [3]. Assuming a mean difference of 2.7 mm (SD 1.97 and 1.84), with 90% power and 5% significance level, a minimum of 12 patients was required. To account for technical issues and dropouts, 19 patients were included.

## 3 Results

**FIGURE 3:**
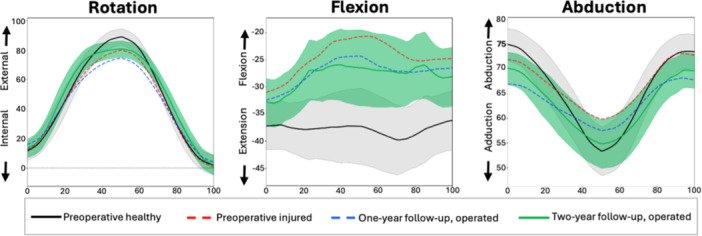
GHJ range of motion in degrees during the external rotation motion. Time point zero on the *x*‐axis represents the starting position in internal rotation, 50 corresponds to the position of maximal external rotation, and 100 marks the return to the end position in internal rotation. The variation of the range of motion for the preoperative healthy shoulder and the operated shoulder at 2‐year follow‐up is depicted with shaded areas representing the 95% confidence intervals.

**FIGURE 4:**
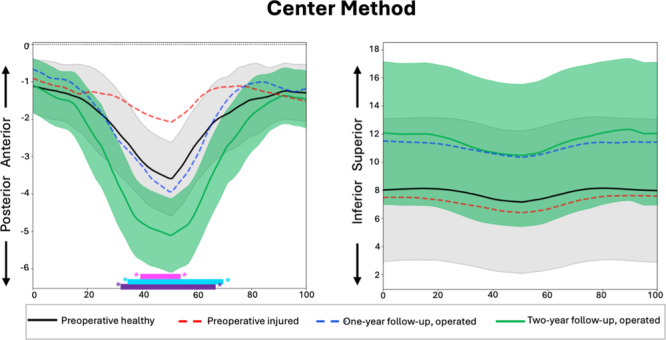
GHJ kinematics (mm) of the humeral head center translations during the external rotation motion for the healthy shoulders and the injured and post‐operative shoulders. Time point zero represents the starting position in internal rotation, 50 corresponds to the position of maximal external rotation, and 100 marks the return to the end position in internal rotation. The preoperative healthy shoulder and the operated shoulder at 2‐year follow‐up are depicted with shaded areas representing the 95% confidence intervals. Statistically significant differences are displayed by a colored bar at the bottom between the preoperative injured shoulder and the operated shoulder at 1‐year follow‐up (purple), between the preoperative injured shoulder and the operated shoulder at 2‐year follow‐up (cyan), and between the 1‐year follow‐up and 2‐year follow‐up of the operated shoulders (pink).

**FIGURE 5:**
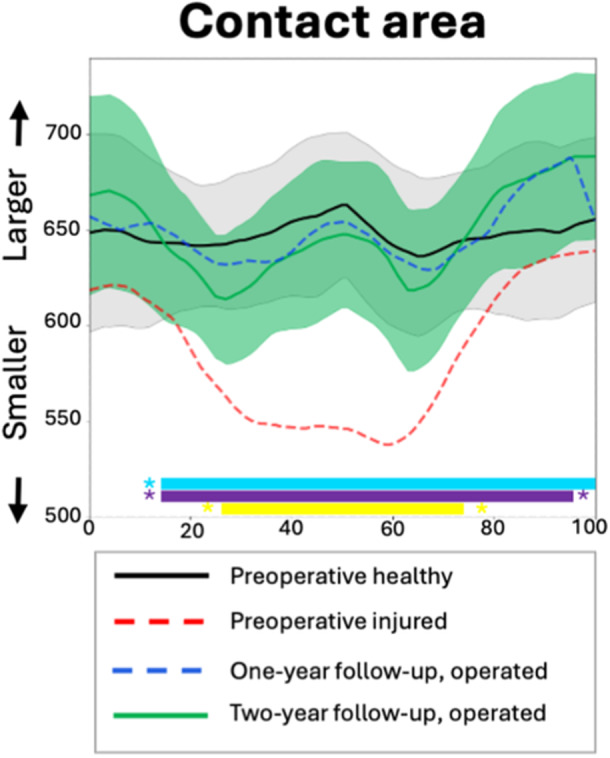
The contact area in mm^2^ during the external rotation motion. Time point zero represents the starting position in internal rotation, 50 corresponds to the position of maximal external rotation, and 100 marks the return to the end position in internal rotation. The preoperative healthy shoulder and the operated shoulder at 2‐year follow‐up are depicted with shaded areas representing the 95% confidence intervals. Statistically significant difference between the preoperative injured shoulder and preoperative healthy shoulder (yellow), the preoperative injured shoulder and the operated shoulder at 1‐year follow‐up (purple), and between the preoperative injured shoulder and the operated shoulder at 2‐year follow‐up (cyan).

The cohort consisted of 19 patients (16 males) with a median age of 28 years (range 21–40), and a mean glenoid bone loss of 17.4% (95% CI 14.6–20.3). All patients completed the 1‐year follow‐up, and three patients were lost to follow‐up at 2 years. Detailed data on range of motion (Figure 3), translations (Figure 4), and contact area (Figure 5) are presented, showing symmetrical patterns across both external and internal rotation for all measures. Supporting Information Tables S1 and S2 show translation and contact area differences. All values reported in the results section are presented as mean maximum values with 95% confidence intervals (95% CI).

### 3.1 Range of Motion

The mean external rotation of the healthy shoulder ranged from 11° (6–16) to 89° (86–92). The injured shoulder demonstrated a reduced range of external rotation, from 12° (7–18) to 80° (77–83), which decreased further at the 1‐year follow‐up to 17° (11–23) to 76° (74–78), but improved at 2 years thus ranging from 13° (7–20) to 81° (79–83). For the flexion angle, the humerus of the injured shoulder was more flexed by 17.5° (9.7–25.2) compared to the healthy shoulder, with no difference between the injured shoulder and 1‐ or 2‐year follow‐up. For the abduction angle, no differences were found between the injured, healthy, and postoperative shoulder at any time point.

### 3.2 Anterior‐Posterior Humeral Head Translations

Preoperatively, the humeral head center of the injured shoulder was up to 1.5 mm (−0.3–3.3) more anteriorly than in the healthy shoulder, particularly at end‐range external rotation. At 1 year postoperatively, the humeral head center of the operated shoulder was more anterior at the start of motion by up to 0.3 mm (−0.4–1.1) and more posterior from mid‐ to end‐range rotation by up to 1.8 mm (0.8–2.9) compared to the preoperative injured shoulder. At 2 years follow‐up, the humeral head center of the operated shoulder was located up to 1.5 mm (0.4–2.6) more posteriorly during mid‐range external rotation than at the 1‐year follow‐up. No statistically significant or clinically relevant differences were found between the operated and healthy shoulder at either follow‐up.

### 3.3 Superior‐Inferior Humeral Head Translation

The humeral head center of the injured shoulder was consistently positioned more inferiorly during motion, by up to 1.1 mm (−0.3–2.5), compared to the healthy side. A nonsignificant trend toward a 4.0 mm (−4.0–12.0) more superior position at 1 year was observed. One year postoperatively, the humeral head center was up to 3.2 mm (−4.9–11.4) more superior than the healthy shoulder. No differences were observed between the 1‐ and 2‐year follow‐ups.

### 3.4 Contact Area

For the injured shoulder, the contact area was decreased by up to 121.7 mm^2^ (57.1–186.3) compared to the healthy shoulder. At the 1‐year follow‐up after the Latarjet procedure, the contact area of the operated shoulder increased by up to 110.8 mm^2^ (42.3–179.3) compared to the injured GHJ. No difference was found between the 1‐year and 2‐year follow‐up, or in comparison to the healthy shoulder.

### 3.5 Patient‐Reported Outcome

The mean preoperative WOSI score was 55% (49–61). At 1‐year follow‐up, the WOSI score improved significantly by 19 percentage points (0–28) to 36% (25–48). At the 2‐year follow‐up, an additional improvement of 8 percentage points (0–15) was observed, resulting in a mean score of 27% (17–36).

## 4 Discussion

This study investigated the GHJ kinematics during dynamic external rotation in patients undergoing the Latarjet procedure. The primary findings were that the postoperative GHJ kinematics were comparable to the healthy shoulder, with a more posteriorly positioned humeral head center and an increased contact area compared to the preoperative injured shoulder.

### 4.1 Kinematic Stabilization After the Latarjet Procedure

The postoperative translation pattern of the humeral head center observed in this study aligns with previous research, suggesting that the Latarjet procedure alters GHJ kinematics, particularly at the end range of external rotation [4, 11]. Park et al., using biplane fluoroscopy, reported that following the Latarjet procedure, the humeral head initially translated anteriorly until 40° of external rotation, followed by a posterior translation by up to 5.27 mm compared to the contralateral side at the end range [4]. Similarly, Giacomo et al., employing magnetic resonance imaging (MRI) in an isometric external rotation position, observed a 3.95 mm posterior displacement in shoulders after the Latarjet procedure as compared to healthy controls [3]. In a cadaveric study using dynamic RSA, Kipp et al. also reported a posterior humeral head position of 5.4 mm at the end range of external rotation in shoulders following the Latarjet procedure, compared with healthy shoulders without an anterior‐directed load applied to the GHJ [12]. These findings have raised concerns that the Latarjet procedure may predispose to excessive posterior translation of the humeral head in the absence of an anteriorly directed load. In the present study, no significant differences were found between the operated and contralateral healthy shoulders in the anterior‐posterior direction during external rotation, suggesting restored joint stability without abnormal posterior displacement. However, external rotation was on average 13° less in the operated shoulder compared with the contralateral healthy shoulder, which may have affected the humeral head translations.

### 4.2 One vs. Two Years Follow‐Up

GHJ kinematics following the Latarjet procedure have been considered stable over time, with clinical evaluations limited to a single postoperative time point, typically 1 year postoperative [3, 4, 5]. Previous studies have reported substantial coracoid graft resorption within the first 2 postoperative years, which could potentially affect GHJ kinematics [5, 29]. To investigate this, the present study assessed GHJ kinematics at both 1‐ and 2‐year follow‐up. At 2 years, the humeral head center was located up to 1.5 mm more posteriorly than at 1 year, suggesting progressive change in joint positioning over time. Part of this increased posterior translation may be attributable to the more externally rotated arm position observed at the 2‐year follow‐up, which would be expected to result in a more posterior humeral head position. Nevertheless, the improvements in both rotational and translational kinematics suggest a progressive development over time. This is further supported by patient‐reported outcomes, as measured by the WOSI, which improved by 8 percentage points between the 1‐ and 2‐year follow‐ups. In addition, the range of external rotation increased by a mean of 4.6° (95% CI 0.6–8.5) over the same period.

### 4.3 The Contact Area

To our knowledge, this is the first clinical study to evaluate the GHJ contact area following the Latarjet procedure. Previous experimental studies reported decreased contact area after injury and restoration following the Latarjet procedure [30, 31, 32]. The findings in this study are consistent with these results, demonstrating restored GHJ contact area at both 1‐ and 2‐year follow‐ups. This observation is particularly notable considering previous reports of substantial coracoid graft resorption at approximately 60% after the procedure, including one study based on the same cohort as the present study [5, 29]. Despite this resorption, GHJ kinematics remained largely restored in our cohort, suggesting that full preservation of the initial graft area may not be necessary for biomechanical stability and may contribute to reduced risk of osteoarthritic changes.

### 4.4 Strengths and Limitations

A major strength of this study is the use of dynamic RSA, which enables highly accurate, three‐dimensional tracking of GHJ kinematics during motion without data loss. The prospective design allowed intra‐individual pre‐ and postoperative comparisons. However, in evaluating the restoration of GHJ kinematics, the study assumes the contralateral healthy shoulder reflects the pre‐injury kinematics of the operated side [33]. Another limitation of this study is the absence of cartilage on the bone model, which may affect the calculation of the GHJ contact area. However, studies have shown a homogeneous cartilage distribution of the humerus and glenoid and given the young age of our cohort with a low risk of osteoarthritis, we assume adding cartilage on the bone models would not significantly change the contact area [34].

## 5 Conclusion

The Latarjet procedure resulted in GHJ kinematics comparable to the contralateral healthy shoulder. Postoperatively, GHJ kinematics continued to exhibit posterior and superior shifts, while WOSI scores improved, indicating progressive enhancements in shoulder function during the first 2 years following the Latarjet procedure.

## Author Contributions

**Josephine Olsen Kipp:** conceptualization, investigation, data curation, formal analysis, project administration, writing – original draft. **Emil Toft Petersen:** conceptualization, data curation, methodology, formal analysis, supervision, writing – review and editing. **Maiken Stilling:** conceptualization, data curation, funding acquisition, methodology, resources, supervision, validation, writing – review and editing. **Anna Zejden:** investigation, resources, writing – review and editing. **Rikke Jellesen Åberg:** investigation, resources, writing – review and editing. **Thomas Falstie‐Jensen:** investigation, data curation, resources, writing – review and editing. **Theis Muncholm Thillemann:** conceptualization, data curation, supervision, resources, investigation, writing – review and editing.

## Supporting information


**Table S1:** GHJ kinematic differences in mm of the humeral head center between the preoperative healthy shoulder, the preoperative injured shoulder, and the operated shoulder at one‐year and two‐year follow‐up. Statistically significant differences are marked with a *. For the minimum and maximum differences, it is stated which part of the rotation cycle they occurred in.
**Table S2:** Contact area differences in mm^2^ between the preoperative healthy shoulder, the preoperative injured shoulder, and the operated shoulder at one‐year and two‐year follow‐up. Statistically significant differences are marked with a *. For the minimum and maximum differences, it is stated where in the rotation cycle the differences were found.

## Data Availability

The data that support the findings of this study are available from the corresponding author upon reasonable request.
