# Supplementary material for: Glenohumeral Stability During External Rotation in Patients Treated With the Latarjet Procedure: A Two‐Year Dynamic Radiostereometric Study
Source: J Orthop Res. 2026 Aug 9;44(8):e70258. doi: 10.1002/jor.70258 (PMC13453918; doi:10.1002/jor.70258)
Supplement: Supplementary file 1 [file JOR-44-0-s001.docx]

|  | **Start (0)** | **Mid (50)** | **End (100)** | **Min** | **Max** |
| --- | --- | --- | --- | --- | --- |
| **Preoperative healthy vs preoperative injured** | | | | | |
| Anterior-posterior translation | -0.6  (-2.2 to 0.9) | 0.6  (-0.6 to 1.7) | -0.4  (-1.8 to 0.9) | 0.0  (-1.4 to 1.4) at 82% | 1.5  (-0.3 to 3.3) at 50% |
| Superior-inferior translation | -0.8  (-1.8 to 0.4) | -0.9  (-2.4 to 0.6) | -0.6  (-1.7 to 0.6) | -0.6  (-1.7 to 0.6) at 96% | -1.1  (-2.5 to 0.3) at 29% |
| **Preoperative injured vs one-year follow-up, injured** | | | | | |
| Anterior-posterior translation | 0.3  (-0.4 to 1.1) | -1.8  (-2.9 to -0.8)* | 0.4  (-0.6 to 1.4) | 0.0  (-0.7 to 0.7) at 76% | -1.8  (-2.9 to -0.8) at 50%* |
| Superior-inferior translation | 4.0  (-4.1 to 12.0) | 3.9  (-4.2 to 11.9) | 3.8  (-4.0 to 12.0) | 3.7  (-4.3 to 11.7) at 85% | 4.0  (-4.0 to 12.0) at 60% |
| **Preoperative injured vs two-year follow-up, injured** | | | | | |
| Anterior-posterior translation | 0.1  (-0.7 to 1.0) | -3.0  (-4.6 to 1.4)* | 0.3  (-0.4 to 1.1) | 0.0  (-0.9 to 0.9) at 7% | -3.0  (-4.6 to 1.4) at 50%* |
| Superior-inferior translation | 4.7  (-5.1 to 14.6) | 4.2  (-5.5 to 13.8) | 4.6  (-5.3 to 14.4) | 4.2  (-5.5 to 13.8) at 46% | 4.8  (-5.1 to 14.6) at 16% |
| **One year follow-up, injured vs two-year follow-up, injured** | | | | | |
| Anterior-posterior translation | -0.2  (1.2 to 0.8) | -1.0  (-2.2 to 0.2) | -0.1  (-1.2 to 0.9) | 0.0  (-0.8 to 0.8) at 97% | -1.5  (-2.6 to -0.4) at 38%* |
| Superior-inferior translation | 0.1  (-0.2 to 0.5) | -0.4  (-1.1 to 0.3) | 0.1  (-0.1 to 0.6) | 0.0  (-0.6 to 0.6) at 74% | -0.5  (-1.1 to 0.1) at 38% |
| **Preoperative healthy vs one-year follow-up, injured** | | | | | |
| Anterior-posterior translation | 0.2  (-1.4 to 1.7) | -0.5  (-2.4 to 1.5) | -0.1  (-1.5 to 1.4) | 0.0  (-1.7 to 1.7) at 68% | -0.5  (-2.3 to 1.3) at 58% |
| Superior-inferior translation | 3.2  (-4.9 to 11.4) | 3.0  (-5.3 to 11.3) | 3.2  (-5.0 to 11.4) | 2.8  (-5.5 to 11.2) at 30% | 3.2  (-4.9 to 11.4) at 0% |
| **Preoperative healthy vs two-year follow-up, injured** | | | | | |
| Anterior-posterior translation | 0.2  (-1.4 to 1.5) | -1.5  (-3.5 to 0.5) | -0.2  (-1.7 to 1.4) | 0.0  (1.4 to 1.5) at 1% | -1.8  (-3.9 to 0.2) at 58% |
| Superior-inferior translation | 4.1  (-5.9 to 14.0) | 3.3  (-6.6 to 13.3) | 4.1  (-5.9 to 14.0) | 3.3  (-6.7 to 13.3) at 37% | 4.1  (-5.9 to 14.0) at 100% |

**Table 1 (supplementary material)** GHJ kinematic differences in mm of the humeral head center between the preoperative healthy shoulder, the preoperative injured shoulder, and the operated shoulder at one-year and two-year follow-up. Statistically significant differences are marked with a *. For the minimum and maximum differences, it is stated where in the rotation cycle the differences were found.

**Table 2 (supplementary material)** Contact area differences in mm^2^ between the preoperative healthy shoulder, the preoperative injured shoulder, and the operated shoulder at one-year and two-year follow-up. Statistically significant differences are marked with a *. For the minimum and maximum differences, it is stated where in the rotation cycle the differences were found.

|  | **Start (0)** | **Mid (50)** | **End (100)** | **Min** | **Max** |
| --- | --- | --- | --- | --- | --- |
| **Preoperative healthy vs preoperative injured** | -23.5  (-91.8 to 44.9) | -121.7  (-186.3 to -57.1)* | -11.8  (-80.2 to 56.1)* | -8.0  (-76.7 to 60.8) at 92 ° | -121.7  (-186.3 to -57.0)* at 50° |
| **Preoperative injured vs one-year follow-up, injured** | 40.0  (-5.3 to 85.3) | 107.9  (56.6 to 169.2)* | 18.4  (-127.3 to 164.2) | 18.4  (-127.3 to 164.2) at 100° | 107.9  (46.6 to 169.2)* at 50° |
| **Preoperative injured vs two-year follow-up, injured** | 55.6  (-18.0 to 129.3) | 109.7  (41.9 to 177.6)* | 56.9  (17.6 to 96.3)* | 43.9  (-25.8 to 113.7) at 16° | 110.8  (42.3 to 179.3)* at 55° |
| **One year follow-up, injured vs two-year follow-up, injured** | 7.8  (-75.8 to 91.3) | -5.3  (-45.8 to 35.2) | 43.3  (-132.4 to 218.9) | -0.1  (-76.7 to 76.6) at 12° | 43.3  (-132.4 to 218.9) at 100° |
| **Preoperative healthy vs one-year follow-up, injured** | 18.4  (-54.5 to 91.4) | -7.8  (-69.3 to 53.7) | 9.2  (-155.6 to 173.9) | 0.1  (-70.7 to 70.8) at 25° | 45.7  (-29.5 to 120.9) at 93° |
| **Preoperative healthy vs two-year follow-up, injured** | 19.5  (-84.4 to 123.4) | -15.4  (-91.6 to 60.8) | 33.1  (-53.2 to 119.3) | 0.0  (-72.4 to 72.5) at 74° | 36.5  (-51.6 to 124.5) at 95° |
